# Iranian Nurses’ Perception of Patient Safety Culture

**DOI:** 10.5812/ircmj.11894

**Published:** 2014-04-05

**Authors:** Mohammad Amin Bahrami, Mahjabin Chalak, Razieh Montazeralfaraj, Arefeh Dehghani Tafti

**Affiliations:** 1 Department of Healthcare Management, Shahid Sadoughi University of Medical Sciences, Yazd, IR Iran; 2 Hospital Management Research Center, Shahid Sadoughi Hospital, Yazd, IR Iran; 3 Shahid Sadoughi University of Medical Sciences, Yazd, IR Iran; 4 Department of Epidemiology and Biostatistics, Shahid Sadoughi University of Medical Sciences, Yazd, IR Iran

**Keywords:** Patients, Patient Safety, Nurses, Hospital

## Abstract

**Background:**

In recent decades, patient safety has become a high priority health system issue, due to the high potential of occurring adverse events in health facilities.

**Objectives:**

This study was aimed to survey patient safety culture in 2 Iranian educational hospitals.

**Materials and Methods:**

In a descriptive, cross-sectional survey, a hospital survey on patient safety culture, was used in two teaching hospitals in Yazd, Iran during 2012. Study population was comprised of the same hospitals' nurses. Stratified-random sampling method was used and distributed among a total of 340 randomly-selected nurses from different units. From all distributed questionnaires, 302 ones were answered completely and afterwards analyzed using SPSS 17. Dimensional- and item-level positive scores were used for results reporting. Additionally descriptive statistics (mean and standard deviation), independent sample t-test and ANOVA were sued for data analyzing.

**Results:**

Research findings demonstrated that both hospitals had low to average scores in all dimensions of patient safety culture. Non-punitive response to error, staffing and frequency of events reported had the lowest positive scores of patient safety dimensions with scores 15.26, 19.26, 16.65, 30 and 32.87, 31.10 respectively in Shahid Sadoughi and Shahid Rahnemoon Hospitals. Also only 29.20 and 28.80 percent of nurses in Shahid Sadoughi and Shahid Rahnemoon Hospitals, respectively, evaluated the patient safety grade of their hospital as “excellent” and “very good”. Indeed, the studied hospitals had a statistical difference in 3 dimensions of patient safety culture (frequency of events reported, organizational learning and staffing). (P ≤ 0.05)

**Conclusions:**

Our study results were indicating of the challenge of weak patient safety culture, in educational hospitals. Therefore, the issue should be integrated to all policy makers and managerial initiatives in our health system, as a top priority.

## 1. Background

Mistake is an inevitable part of the human life (1). Although, errors occur in all kinds of works, but working in healthcare organizations is more error-prone due to the complexity of this area (2). Recent reports indicate that too many evitable errors and harms occur in healthcare facilities, threatening the life of millions of patients around the world. Therefore in last decades, reducing the errors in care process and improvement of care quality and patient safety have become high priority health system issues and have frequently caused controversies between health policy makers and managers (3). The 2000 report of U.S. institute of medicine intensified these controversies (4). Improvement of patient safety, in terms of risks and outcomes, in a healthcare system depends on the building of patient safety culture (5). Therefore a responsible safety culture should be developed as an essential element of any effort in search of promoting the health services quality and patient safety (6). Generally, culture refers to a set of shared values, assumptions, traditions, norms, beliefs, attitudes and principles, respected by a community. Therefore, patient safety culture, can be defined as the same concepts in relation to patient safety. In the other words, safety culture, a subset of organizational culture, means the integration of safety thinking and practicing with clinical practices in healthcare (5, 7, 8), influenced by numerous factors, collectively named safety dimensions, domains or affecting factors. These factors could be summarized as following:

1. Organizational factors like organizational preparation and commitment towards redesigning and continuous learning.
2. Work environment factors like preparing blame-free environment where errors are readily reported and served as a source of continuous improvement.
3. Team-building and teams like developing team-building skills inside the organization and effective communication between individuals.
4. Resources/staffing and individual factors like enough staffing, honesty, respect, competencies, job commitment, job satisfaction and motivation (9, 10).

Creating the safety culture is a challenging issue due to requiring substantial changes in the basic concepts of organizational life. Also, until now, there are no standardized methods to guide health professionals in the road of developing similar cultures. Indeed, the resource shortage in health systems, weakens their ability of preparing the requirements of a strong patient safety culture (9). Nevertheless, based on the international patient safety strategies, assessment of patient's culture could be implemented as an effective strategy in improvement initiatives. Therefore assessing the patient safety culture can be spotted as one of the first steps in safety promotion, because understanding of the current situation is helpful for developing effective safety promotion plans and policies (11). Indeed, assessing the patient safety culture has also other benefits: it helps detecting vulnerabilities and evaluating improvement initiatives to strengthen them. Additionally these results' assessment, can hopefully be used for benchmarking purposes (12).

The ongoing dispute in the safety science about the terms culture, climate and attitude is notable. Although, these terms have been used synonymously in some literatures, some authors believe they represent different concepts. They believe the term culture is more subjective and invisible than climate, therefore they believe that the safety climate can be thought as the quantitative manifestation of the safety culture, which can be quantified with visible indicators like outcome (like the number of adverse events) or process indicators (like the rate of adherence to caring process guidelines). Despite this, due to the subjective nature of culture, most of the time, the safety climate measures are assessed, as a proxy of the safety culture. Safety attitude is another used term in the safety literature but its definitions are clearer than that of the safety climate. Safety attitude has an individual level of concept. It is an individual attribute describing the individual attitudes about safety. In this study, while acknowledging of the conceptual differences between these terms, given the instrument used for data gathering, the term safety was selected culture (6, 7, 10, 13).

Based on the available knowledge and experience, little is known about the patient safety culture in educational hospitals in Iran. Assuming that measuring patient safety culture can be implemented as an initial step in improving patient safety and outcomes, this study was undertaken to assess the patient safety culture, in two educational hospitals in Yazd, Iran. Our goal was to survey the safety culture throughout these hospitals to improve the discussion about this necessity in the healthcare context, in addition to promote the patient safety culture in studied hospitals.

## 2. Objectives

This research was aimed to assess the safety culture in two sample educational hospitals, in order to enhance the awareness and awake challenges about this necessity in the entire work environment of Iranian hospitals.

## 3. Materials and Methods

The purpose of this study was to assess the Iranian nurses' perception of safety culture in two Iranian educational hospitals. The data presented here were collected from two hospitals (Shahid Sadoughi and Shahid Rahnemoon both general hospitals affiliated to Shahid Sadoughi University of Medical Sciences) Yazd, Iran, during 2012. Data were collected using Hospital Survey on Patient Safety Culture (HSOPSC). The HSOPSC consists of 42 items, categorized in 12 dimensions. The survey measures seven unit-level aspects of safety culture including: supervisor/manager expectations & promoting safety actions (four items), organizational learning ([continuous improvement], [three items]), teamwork within units (four items), communication facilities (3 items), feedback and communication about errors (3 items), non-punitive response to errors (3 items) and staffing (4 items). In addition, the survey measures three hospital-level aspects of safety culture: hospital management support for patient safety (3 items), teamwork across hospital units (4 items) andhospital handoffs and transitions (4 items). Finally, two outcome variables were included: overall perceptions of safety (4 items) and frequency of event reporting (3 items) (14-18).

In a descriptive, cross-sectional study, a total of 340 paper-based questionnaires were distributed among randomly-selected nurses from different units of hospitals. Stratified-random sampling method was used. From all distributed surveys, 302 ones (189 from Shahid Sadoughi Hospital and 113 from Shahid Rahnemoon Hospital) were answered completely and analyzed with a response rate of 89%. The sample size was calculated trough Cochran's formula by using online sample size calculation software, with a confidence level of 95%. The high response rate was due to the reminder letters, sent to participants, by authors. In the first survey a cover letter was also sent with the main questionnaire. The Persian version of HSOPSC which has been validated by Amiresmaili et al. was used in the present study. (14). Prior to the study, the questionnaire’s reliability was obtained with Cronbach's alpha as 0.84, in a pilot study with 34 samples in the studied hospitals. All samples were informed that the collected data were kept confidentially. The study was also approved by Shahid Sadoughi University of Medical Sciences with no. p/17/1/40633. Data analysis was done using SPSS software English version 17. Dimensional- and item- level average percent and percent of positive scores were used for results reporting. In HSOPSC tool, the respondents are questioned to indicate their agreement or disagreement with the questionnaire statements about their work area / unit in 5-points Likert scale (strongly disagree to strongly agree or never to always). The positive scores for each item is defined as the percentage of strongly agree and agree (or always and most of the time) responses for directly-worded items and strongly disagree and disagree (or never and rarely) for reverse-worded items. Also, the average percentage of positive scores for each level, is defined as the mean of positive scores per cents for that dimension’s related items. Indeed, descriptive statistics (mean and standard deviation), independent sample t-test and ANOVA test were used in data analyzing. In this study, after positive scores calculation, (average) percent for each dimension and item, the results were compared with those from other reported studies. It is worth mentioning that incomplete surveys (surveys with no entire section completed, all items answered the same or fewer than half items answered) were removed, before the data analysis.

## 4. Results

The characteristics of sample are shown in Table 1. Also the dimensional scores of hospitals and comparison with some previous surveys results are shown in the Table 2 and the mean scores of patient safety culture dimensions in different units of hospitals and the differences between them based on the hospital and some demographic features of respondent are presented in Tables 3-5.

**Table 1.** Characteristics of Subjects ^a^

	A ^b^	B ^b^
**Work area/unit:**		
Internal (medical) unit	29 (15)	33 (29)
Surgical unit	26 (14)	23 (20)
Obstetrics and gynecology	13 (7)	30 (27)
Pediatrics	26 (14)	15 (13)
Neurology	8 (4)	12 (11)
Intensive care	26 (14)	
Emergency department	13 (7)	
Other	48 (25)	
**Professional experience, y**		
< 1	26 (14)	18 (16)
1-5	45 (24)	33 (29)
6-10	65 (34)	18 (16)
11-15	45 (24)	25 (22)
≥ 16	8 (4)	19 (17)
**Professional experience in the same unit, y**		
< 1	39 (21)	28 (24)
1-5	81 (43)	43 (38)
6-10	37 (19)	30 (27)
≥ 11	32 (17)	12 (11)
**Professional experience in the same position, y**		
< 1	18 (10)	12 (11)
1-5	47 (25)	37 (32)
6-10	53 (28)	26 (23)
11-15	47 (25)	38 (34)
≥ 16	24 (12)	0
**Working time in hospital, hours per week**		
< 20	6 (3)	0
20-39	34 (18)	7 (6)
40-59	115 (61)	88 (78)
≥ 60	34 (18)	18 (16)

^a^ Data are presented in No. (%).

^b^ A: Shahid Sadoughi Hospital, B: Shahid Rahnemoon Hospital.

**Table 2.** Dimensional- and Item- Level Frequency (Percent) of Positive Scores, 2012 ^a^

Dimensions and Items	Positive Scores				
	Shahid Sadoughi Hospital- 2012	Shahid Rahnemoon Hospital-2012	AHRQ 2012 Report (15)	Afshar Hospital- Iran, 2012 (16)	Firoozgar Hospital- Iran, 2008 (14)
**Overall perceptions of safety**	94 (49.70)	57 (50.40)	66.00	66.22	59.50
Patient safety is never sacrificed to get more work done. ^b^	126 (66.70)	93 (82.20)	62.00	77.80	NR
Our procedures and systems are good for preventing errors from happening. ^b^	87 (45.80)	33 (28.90)	64.00	44.40	NR
It is just by chance that more serious mistakes do not occur. ^c^	123 (65.30)	68 (60.00)	64.00	88.90	NR
We have patient safety problems in this department. ^c^	39 (20.80)	33 (28.90)	72.00	37.80	NR
**Frequency of events reported**	62 (32.87)	35 (31.10)	63.00	34.90	50.17
When a mistake is made but is caught and corrected before affecting the patient, how often is this reported? ^d^	92 (48.60)	48 (42.20)	57.00	55.60	NR
When a mistake is made but has no potential to harm the patient, how often is this reported? ^d^	53 (27.80)	30 (26.70)	59.00	24.40	NR
When a mistake is made that could harm the patient but does not, how often is this reported? ^d^	42 (22.20)	28 (24.40)	74.00	24.40	NR
**Supervisor/manager expectations & actions promoting patient safety**	83 (44.07)	41 (36.67)	75.00	36.12	70.00
My supervisor/manager says a good word when he/she sees a job done according to established patient safety procedures. ^b^	45 (23.60)	20 (17.80)	73.00	15.60	NR
My supervisor/manager seriously considers staff suggestions for improving patient safety. ^b^	68 (36.10)	40 (35.60)	76.00	26.70	NR
Whenever pressure builds up, my supervisor/manager wants us to work faster, even if it means taking shortcuts. ^c^	84 (44.40)	48 (42.20)	74.00	40.00	NR
My supervisor/manager overlooks patient safety problems happening over and over. ^d^	136 (72.20)	58 (51.10)	76.00	62.20	NR
**Organizational learning –continuous improvement**	122 (64.37)	64 (56.30)	72.00	71.86	66.90
We are actively doing things to improve patient safety. ^b^	150 (79.20)	95 (84.40)	84.00	86.70	NR
Mistakes have led to positive changes here. ^b^	87 (45.80)	30 (26.70)	64.00	48.90	NR
After we make changes to improve patient safety, we evaluate their effectiveness. ^b^	129 (68.10)	65 (57.80)	69.00	80.00	NR
**Teamwork within units**	87 (46.17)	73 (65.00)	80.00	68.87	71.40
People support one another in this department. ^b^	142 (75.00)	75 (66.70)	86.00	80.00	NR
When a lot of work needs to be done quickly, we work together as a team to get the work done. ^b^	126 (66.70)	98 (86.70)	86.00	82.20	NR
In this department, people treat each other with respect. ^b^	18 (9.70)	85 (75.60)	78.00	84.40	NR
When one area in this department gets really busy, others help out. ^b^	63 (33.30)	35 (31.10)	69.00	28.90	NR
**Communication openness**	71 (37.50)	42 (37.10)	62.00	37.06	60.00
Staff will freely speak up if they see something that may negatively affect patient care. ^b^	81 (43.10)	53 (46.70)	75.00	57.80	NR
Staff feel free to question the decisions or actions of those with more authority. ^b^	13 (6.90)	25 (22.20)	47.00	6.70	NR
Staff are afraid to ask questions when something does not seem right. ^c^	118 (62.50)	48 (42.20)	63.00	46.70	NR
**Feedback and communication about error**s	63 (33.36)	37 (32.60)	64.00	33.56	64.80
We are given feedbacks about changes put into place based on event reports. ^b^	79 (41.70)	30 (26.70)	56.00	40.00	NR
We are informed about errors happening in this department. ^b^	81 (43.10)	53 (46.70)	65.00	48.90	NR
In this department, we discuss ways to prevent reoccurring errors. ^b^	29 (15.30)	28 (24.40)	72.00	17.80	NR
**Non-punitive response to error**	29 (15.30)	22 (19.26)	44.00	21.46	22.80
Staff feel like their mistakes are held against them. ^b^	45 (23.60)	20 (17.80)	50.00	24.40	NR
When an event is reported, it feels like the person is being written up, not the problem. ^c^	37 (19.40)	35 (31.10)	46.00	28.90	NR
Staff worry that mistakes they make are kept in their personnel file. ^c^	5 (2.80)	10 (8.90)	35.00	11.10	NR
**Staffing**	31 (16.65)	34 (30.00)	56.00	19.45	38.10
We have enough staff to handle the workload. ^b^	37 (19.40)	30 (26.70)	56.00	17.80	NR
Staff in this department work longer hours than is best for patient care. ^c^	11 (5.60)	25 (22.20)	53.00	11.10	NR
We use more agency/temporary staff than is best for patient care. ^c^	63 (33.30)	68 (60.00)	68.00	35.60	NR
We work in “crisis mode,” trying to do too much, too quickly. ^c^	16 (8.30)	13 (11.10)	50.00	13.30	NR
**Hospital management support for patient safety**	95 (50.43)	50 (44.40)	72.00	37.00	32.20
Hospital management provides a work climate that promotes patient safety. ^b^	110 (58.30)	50 (44.40)	81.00	44.40	NR
The actions of hospital management show that patient safety is the top priority. ^b^	115 (61.10)	73 (64.40)	75.00	42.20	NR
Hospital management seems interested in patient safety only after an adverse event happens. ^c^	60 (31.90)	28 (24.40)	61.00	24.40	NR
**Teamwork across hospital units**	77 (40.62)	46 (41.12)	58.00	55.55	43.8
There is good cooperation among hospital departments that need to work together. ^b^	89 (47.20)	40 (35.60)	46.00	62.20	NR
Hospital departments work well together to provide the best care for patients.	97 (51.42)	65 (57.80)	60.00	66.70	NR
Hospital departments do not coordinate well with each other. ^c^	42 (22.20)	30 (26.70)	59.00	24.40	NR
It is often unpleasant to work with staff from other hospital departments. ^c^	79 (41.70)	50 (44.40)	68.00	68.90	NR
**Hospital handoffs & transitions**	97 (51.42)	60 (53.35)	45.00	58.35	54.20
Things “fall between the cracks” when transferring patients from one department to another. ^c^	53 (27.80)	40 (35.60)	41.00	35.60	NR
Important patient care information is often lost during shift changes. ^c^	129 (68.10)	78 (68.90)	51.00	82.20	NR
Problems often occur during exchange of the information across hospital departments. ^c^	81 (43.10)	38 (33.30)	44.00	48.90	NR
Shift changes are problematic for patients in this hospital. ^c^	126 (66.70)	85 (75.60)	45.00	66.70	NR
**Patient safety grade**	-	-	-	-	-
Excellent	11 (5.60)	5 (4.40)	30.00	11.10	NR
Very good	45 (23.60)	28 (24.40)	45.00	31.10	NR
Acceptable	87 (45.80)	38 (33.30)	20.00	51.10	NR
Poor	29 (15.30)	23 (20.00)	4.00	0.00	NR
Failing	5 (2.80)	15 (13.30)	1.00	6.70	NR
**Number of events reported ** ^e^	-	-	-	-	-
No events report	139 (73.60)	73 (64.40)	55.00	71.10	NR
1 to 2 event reports	32 (16.70)	23 (20.00)	27.00	22.20	NR
3 to 5 event reports	16 (8.30)	8 (6.70)	12.00	6.60	NR
6 events or more reports	00 (0.00)	5 (4.40)	7.00	0.00	NR

^a^ Data are presented as No. (%).

^b^ "Agree" and "strongly agree" are positive responses.

^c^ "Strongly disagree" and "disagree" are positive responses.

^d^ "Most of the times" and "always" are positive responses.

^e^ The "number of events reported" item in 2005, asked respondents how many medication safety reports they have filled out and submitted. The same item in 2007 asked respondents how many event reports they have filled out and submitted.

**Table 3.** Mean and SD of Patient Safety Culture Dimension Scores in Different Units, 2012 ^a^, ^b^

	Internal	Surgery	obstetrics and gynecology	Pediatrics	Mental health	Intensive care	Emergency	Laboratory	Radiology	Other	P Value
**Overall perceptions of safety**	3.21 ± 0.54	2.84 ± 0.74	3.53 ± 0.65	3.07 ± 0.35	3.14 ± 0.47	3.08 ± 0.50	3.46 ± 0.45	3.50 ± 0.43	3.75 ± 0.00	3.18 ± 0.58	0.06
**Frequency of events reported**	3.35 ± 0.89	2.62 ± 0.79	2.95 ± 1.11	2.80 ± 0.68	3.00 ± 0.76	2.92 ± 0.52	3.45 ± 0.30	2.88 ± 0.69	3.00 ± 0.00	2.86 ± 0.83	0.16
**Supervisor/manager expectations & actions for promoting patient safety**	2.77 ± 0.78	2.38 ± 0.72	3.09 ± 0.65	2.30 ± 0.63	2.00 ± 0.72	2.50 ± 0.50	2.81 ± 0.56	3.00 ± 0.25	3.75 ± 0.00	2.30 ± 0.68	0.01 ^c^
**Organizational learning (continuous improvement)**	3.61 ± 0.65	3.15 ± 0.71	3.62 ± 0.48	3.53 ± 0.63	3.33 ± 0.66	3.50 ± 0.46	3.75 ± 0.52	3.55 ± 0.38	4.33 ± 0.57	3.71 ± 0.50	0.04 ^c^
**Teamwork within units**	3.28 ± 0.87	3.07 ± 0.89	3.28 ± 0.75	3.57 ± 0.67	3.28 ± 0.54	3.66 ± 0.58	3.71 ± 0.41	3.75 ± 0.25	3.50 ± 0.00	3.83 ± 0.61	0.08
**Communication openness**	2.71 ± 0.78	2.59 ± 0.76	2.75 ± 0.34	2.56 ± 0.27	3.80 ± 0.74	2.71 ± 0.63	3.33 ± 0.69	3.00 ± 0.57	3.77 ± 0.19	2.42 ± 0.79	0.04 ^c^
**Feedback and communication about error**	3.12 ± 0.55	2.66 ± 0.87	3.28 ± 0.40	3.10 ± 0.49	2.95 ± 0.35	3.14 ± 0.55	3.71 ± 0.59	3.33 ± 0.33	4.33 ± 0.00	3.19 ± 0.48	0.00 ^c^
**Non-punitive response to error**	3.82 ± 0.63	3.65 ± 0.89	4.14 ± 1.18	3.23 ± 0.49	3.85 ± 0.57	3.61 ± 0.76	3.37 ± 0.60	3.66 ± 0.00	4.66 ± 0.00	3.60 ± 69	0.12
**Staffing**	3.25 ± 0.61	3.12 ± 0.73	3.32 ± 0.64	3.30 ± 0.46	3.64 ± 0.55	3.25 ± 0.68	3.40 ± 0.53	3.58 ± 0.14	4.25 ± 0.00	3.36 ± 0.60	0.22
**Hospital management support for patient safety**	3.56 ± 0.78	3.04 ± 0.91	3.03 ± 0.84	3.03 ± 0.33	3.47 ± 0.37	3.26 ± 0.73	3.45 ± 0.85	3.44 ± 0.96	2.66 ± 0.00	3.37 ± 0.78	0.35
**Teamwork across hospital units**	3.22 ± 0.47	3.00 ± 0.62	3.22 ± 0.26	3.20 ± 0.45	3.14 ± 0.40	3.21 ± 0.54	3.21 ± 0.52	3.58 ± 0.14	3.00 ± 0.00	3.15 ± 0.58	0.80
**Hospital handoffs & transitions**	2.93 ± 0.85	2.75 ± 0.71	2.69 ± 0.98	2.22 ± 0.77	2.78 ± 0.90	2.76 ± 0.68	2.75 ± 0.93	3.08 ± 1.25	4.50 ± 0.00	1.82 ± 0.75	0.00 ^c^

^a^ Data are presented in Mean ± SD.

^b^ ANOVA Test.

^c^ Significant at P ≤ 0.

**Table 4.** Differences Between Patient Safety Culture Dimensions Scores in Studied Hospitals, 2012 ^a^, ^b^

	Shahid Sadoughi	Shahid Rahnemoon	P Value
**Overall perceptions of safety**	3.15 ± 0.52	3.16 ± 0.69	0.96
**Frequency of events reported**	3.07 ± 0.71	2.78 ± 0.84	0.05 ^c^
**Supervisor/manager expectations & actions for promoting patient safety**	2.57 ± 0.77	2.53 ± 0.62	0.81
**Organizational learning (continuous improvement)**	3.61 ± 0.55	3.38 ± 0.68	0.05 ^c^
**Teamwork within units**	3.46 ± 0.74	3.43 ± 0.75	0.85
**Communication openness**	2.63 ± 0.64	2.86 ± 0.78	0.09
**Feedback and communication about error**	3.16 ± 0.57	3.03 ± 0.79	0.29
**Non-punitive response to error**	3.73 ± 0.66	3.60 ± 0.88	0.40
**Staffing**	3.47 ± 0.56	3.09 ± 0.65	0.00 ^c^
**Hospital management support for patient safety**	3.30 ± 0.74	3.20 ± 0.81	0.47
**Teamwork across hospital units**	3.20 ± 0.50	3.10 ± 0.50	0.32
**Hospital handoffs & transitions**	2.68 ± 0.93	2.65 ± 0.84	0.86

^a^ Data are presented in Mean ± SD.

^b^ Independent sample t-test.

^c^ significant at P ≤ 0.05.

**Table 5.** Differences Between Scores of Patient Safety Culture Dimensions Based on the Respondent Demographic Features, 2012 ^a^

	P Value			
	Professional Experience, y	Professional Experience in the Same Unit, y	Professional Experience in the Same Position, y	Working Time in Hospital, Hours Per Week
**Overall perceptions of safety**	0.13	0.21	0.22	0.09
**Frequency of events reported**	0.37	0.33	0.22	0.95
**Supervisor/manager expectations & actions for promoting patient safety**	0.27	0.49	0.11	0.34
**Organizational learning (continuous improvement)**	0.08	0.77	0.11	0.51
**Teamwork within units**	0.26	0.03 ^b^	0.03 ^b^	0.60
**Communication openness**	0.00 ^b^	0.01 ^b^	0.00 ^b^	0.56
**Feedback and communication about error**	0.13	0.04 ^b^	0.10	0.61
**Non-punitive response to error**	0.19	0.03 ^b^	0.49	0.11
**Staffing**	0.02 ^b^	0.71	0.30	0.73
**Hospital management support for patient safety**	0.33	0.57	0.07	0.40
**Teamwork across hospital units**	0.19	0.17	0.04 ^b^	0.49
**Hospital handoffs & transitions**	0.98	0.95	0.54	0.01 ^b^

^a^ ANOVA Test

^b^ Significant at P ≤ 0.05.

## 5. Discussion

Our survey indicated that the studied hospitals safety culture scores were in low and in few dimensions, in average rate. Therefore, urgent and imperative action for improving the current situation is inevitable. The results of the present survey showed these hospitals should improve their attitude towards patient safety culture by implementing actions that support all dimensions of a positive safety culture. As shown in Table 2, similar least dimensional scores of patient safety culture in Shahid Sadoughi and Shahid Rahnemoon Hospitals are:

1. Non-punitive response to errors: in this dimension both hospitals are in a weak situation with lowest scores. This means that staff mistakes and the events' reports is held against them, which should not happen (10, 14-20). To solve this problem, Iranian healthcare context requires to avoid the culture “name, shame, blame” and implement the “system approach” in which, each error is viewed as an opportunity to prevent future errors and mistakes and develop protocols and deterrent principles for punitive response.
2. Staffing: these hospitals' scores in staffing demonstrates that there are not enough staff to handle the workload appropriately to provide the qualified care for patients. On the other hand, it shows that these hospitals suffer from staff shortage (10, 14-20). Today, an important challenge of Iranian hospitals is lack of enough staff in contrast with high demand, leading to inappropriate levels of care quality. This issue is of more importance in educational hospitals because their budgets are allocated from the public funds. This limited budgets damage the hospitals potential to recruit enough staff. Also, due to low salaries and benefits of governmental hospitals, most specialists have low motivation to work in these facilities and prefer private practice. Indeed, low financial efficiency of Iranian governmental hospitals decreases their potential to give suitable rewards and benefits to their staff. Therefore, staffing challenge, remains a problem in Iranian hospitals which can harm the patient safety culture and leads to inappropriate levels of care quality.
3. Frequency of events reported: getting a low score in frequency of events reported means mistakes are not reported in these hospitals (10, 14-20). A reporting culture is one in which all members readily report errors and near misses (9). It seems that low frequency of events reported in these hospitals are related to:
  1. Staff and managerial obstacles
  2. Cultural and structural dimensions
  3. Existing punitive response to errors
  4. Lack of excellent clinical incident report system
  5. Poor motivation of specialties for error report due to concerns about their public reputation, especially in private sector.

Our findings showed that Shahid Sadoughi and Shahid Rahnemoon Hospitals have a neutral situation (average score) only in dimensions three and four, respectively. Organizational learning (continuous improvement) and hospital hands off and transition are in average range in both hospitals. Also, in Shahid Sadoughi Hospital, management support for patient safety rated as average. Overall perception of patient safety and teamwork within units has a same situation in Shahid Rahnemoon Hospital. This study demonstrated the challenge of patient safety culture in teaching hospitals. It confirmed that no excellent situation is present in patient safety culture dimensions. These results showed that number of events reported in Shahid Sadoughi and Shahid Rahnemoon Hospitals in 90.3 and 84.4 percent of cases were below two events. Additionally only, 29.20 and 28.80 percent of nurses rated the patient safety grade of their hospital as “excellent” and “very good”. Indeed, dimensional scores found in this study, were lower comparing these results with other surveys including AHRQ 2012 report from US hospitals (15), the survey of patient safety culture in Afshar (16) and Firoozgar Hospitals (14) ( Iranian teaching hospitals located in Yazd and Tehran) and the same study of 24 CAHS of US hospitals in 2007 (19). Therefore it can be concluded that Iranian hospitals are far from a strong positive patient safety culture and changes are inevitable. Based on existing literature and available knowledge and experience in hospital management, some initiatives as following can help promote the culture of safety in Iranian educational hospitals:

1. Establishment, development and optimizing data collection and reporting systems and developing long-term, mid-term and short-term evidence-based programs for improving culture of patient safety in educational hospitals.
2. Focus on the system and avoid individualism in the work environment.
3. Reducing individual blame, through developing rich protocols for avoiding this attitude in case of errors.
4. Increasing negotiations and set suitable rules in national level for recruiting enough healthcare personnel.
5. Development of motivational benefits and incentive packages for health workers.
6. Establishment of patient safety committee in hospitals.
7. Change in educational system through including patient safety courses, in medical universities curriculums. Also, in-service education courses can stress on training patient safety aspects, like communicational skills, data collection and reporting skills and many more.
8. Routine measurement of patient safety for detecting the weaknesses, taking trends in patient safety aspects and planning to improve, are practices to be proposed. Also, assessment results can be integrated in accreditation of hospitals, an action now being performed with medical universities for grading hospitals.
9. And finally, the top managers of healthcare supports are fundamental requirement of patient safety improvement. Development of a national agency or a unit of patient safety in Ministry of Health and Medical Education, in charge of coordinating all initiatives of patient safety improvement in hospitals, is suggested by the authors. Also, non-governmental and civil society organizations can create popular campaigns for supporting patient safety improvements. This study had some strengths and limitations. The strengths of it included the use of a valid survey tool and the high rate of response to questionnaires. Despite these strengths, the study had also some limitations: It was a cross-sectional study, therefore the results of the research cannot be generalized to other hospitals. Additionally the cross sectional studies analyze the data gathered in one point of time, thus indicating the continuous improvement efforts outcomes is not possible through them. Another limitation is that the hospital worker groups include both clinical and non-clinical staff. In this research, only nurses were included as the study population because of their direct contact with the patients. Indeed, a quantitative method was used to assess safety culture. As mentioned in background section, the culture is a subjective concept. Therefore, there are some debts about the usage of quantitative methods (or tools) for measuring a dynamic and complex attribute like culture (21). For solving this problem, some authors have suggested qualitative research methods like focus groups and interviews as more suitable designs for assessing the safety culture (11, 22). Same doubts are relevant to this study.
